# Common cause versus dynamic mutualism: Insights into ADHD's common comorbidities

**DOI:** 10.1002/jcv2.70142

**Published:** 2026-06-18

**Authors:** Zheyue Peng, Ashley L. Watts

**Affiliations:** ^1^ Department of Psychological Sciences Vanderbilt University Nashville Tennessee USA

**Keywords:** ADHD classification, common cause model, comorbidity, dynamic mutualism, externalizing spectrum, neurodevelopmental spectrum

## Abstract

**Background:**

Contemporary classification systems differ in their conceptualization of ADHD as either an externalizing or neurodevelopmental condition, with both perspectives promoting the common cause model as an explanation of ADHD's comorbidity. An alternative theory, dynamic mutualism, proposes that ADHD's comorbidity emerges through reciprocal interactions among symptoms over time, as opposed to a common cause.

**Methods:**

We tested the common cause and the dynamic mutualism theories in explaining the associations between aspects of ADHD (i.e., ADHD, inattention, hyperactivity/impulsivity, cognitive disengagement) and the neurodevelopmental and externalizing spectra across four waves of the Adolescent Brain Cognitive Development (ABCD) Study data (*n* = 11,878 youths aged 9–10 at baseline; 48% female).

**Results:**

Results largely supported the common cause theory in explaining the developmental links between ADHD and both the externalizing and neurodevelopmental spectra. Findings varied across ADHD subdimensions, although not substantially, with hyperactivity/impulsivity more closely linked to the externalizing spectrum and cognitive disengagement more closely linked to the neurodevelopmental spectrum.

**Conclusion:**

ADHD is best conceptualized as a disorder that bridges both the externalizing and neurodevelopmental domains, rather than fitting exclusively within either category. Given the assessment structure of the ABCD Study, there was more support for the common cause model compared with dynamic mutualism.

## Key points

**What's known?**

- Contemporary psychopathology classification systems assume that covariation among conditions within the same category reflects shared etiology, consistent with the common cause model. Alternatively, covariance may be explained by symptom reinforcement, or dynamic mutualism.

**What's new?**

- This study is the first to compare the common cause model and dynamic mutualism in explaining the co‐development of ADHD and other forms of psychopathology to inform ADHD classification.
- The authors found more support for the common cause model compared with dynamic mutualism.
- Their findings support conceptualizing ADHD as bridging the externalizing and neurodevelopmental spectra, rather than fitting exclusively under either spectrum.

**What's relevant?**

- Future studies should aim to identify transdiagnostic risk factors shared between ADHD and externalizing or neurodevelopmental psychopathology as targets for early prevention and intervention.

## INTRODUCTION

Contemporary psychopathology classification systems differ in their conceptualization of ADHD. The DSM‐5‐TR (Diagnostic and Statistical Manual of Mental Disorders, Fifth Edition, Text Revision; American Psychiatric Association, 2013) and ICD‐10 (International Classification of Diseases, 10th Revision; World Health Organization, 2022) position ADHD under neurodevelopmental conditions alongside disorders that are marked by cognitive deficits and generally onset early in development (e.g., autism spectrum disorder, specific learning disorder, and intellectual disabilities; American Psychiatric Association, 2013; Thapar et al., 2017; World Health Organization, 2022). In contrast, the Hierarchical Taxonomy of Psychopathology (HiTOP; Kotov et al., 2021), a modern dimensional classification system of psychopathology, places ADHD under its externalizing spectrum alongside conditions that are characterized by poor behavioral and emotional control (e.g., oppositional defiant disorder, conduct disorder, substance use disorders; Beauchaine et al., 2017; Krueger et al., 2021). Framing ADHD as a neurodevelopmental condition underscores its putative early emerging and underlying neurocognitive deficits (e.g., memory, language ability, psychomotor functions), whereas framing it as an externalizing condition suggests it is primarily a disorder of disinhibition or impulsivity. Substantial evidence supports both placements in competing classification systems (Du Rietz et al., 2021; Michelini et al., 2019; Noordhof et al., 2015; Peng et al., 2025; Pettersson et al., 2023).

### The common cause model of ADHD's comorbidity

Despite these systems' disagreement, both the DSM and HiTOP gesture toward support for the common cause model to explain ADHD's common comorbidities. That is, overlap between two putatively distinct conditions is thought to arise from shared etiologic processes or mechanisms (Beauchaine & McNulty, 2013; Pettersson et al., 2013; Spearman, 1961). For instance, Krueger et al. (2005) noted that comorbidity among externalizing disorders reflects a “common liability [that] underlies expression of these disorders in a single externalizing spectrum,” and Pettersson et al. (2013) described their neurodevelopmental factor as “tapping a broad liability to suffer from a wide range of nonspecific [neurodevelopmental] symptoms” (see also Du Rietz et al., 2021).

The frequent diagnostic co‐occurrence of ADHD and other externalizing disorders (e.g., Angold et al., 1999; Burke et al., 2001; Capusan et al., 2019; Costello et al., 2003; Lee et al., 2011) has been argued to stem from individual differences in disinhibition (Krueger et al., 2021), impulsivity (Beauchaine et al., 2017), or reward processing and self‐control (Neuhaus & Beauchaine, 2017). In short, impulsivity (and related constructs) is thought to serve as a core vulnerability that is shared among externalizing conditions, which sequentially unfold across development through an ontogenic process (Beauchaine et al., 2017). According to this model, impulsivity first manifests as symptoms of ADHD around preschool age. Over time, particularly when ADHD is exacerbated by environmental risk factors (e.g., negative parenting, negative peer influences), ADHD is thought to progress into increasingly severe forms of externalizing psychopathology, namely oppositional defiant disorder, conduct disorder, substance use disorder, and ultimately antisocial personality disorder.

This proposed common cause explanation of ADHD and other externalizing conditions is supported by at least three lines of evidence. First, youth diagnosed with ADHD often engage in impulsive behaviors across development, including aggression, disobedience, substance use, self‐injury, and suicidality (Beauchaine et al., 2010; Burt et al., 2001; Carragher et al., 2014; Hinshaw et al., 2012). Second, longitudinal studies have shown that features of disinhibition (e.g., low effortful control, high surgency) assessed in childhood and adolescence are prospectively predictive of externalizing disorders, including ADHD, conduct disorder, substance use disorders, and antisocial personality disorder (e.g., Elkins et al., 2006; Kirisci et al., 2006; Krueger, 1999a, 1999b; Martel et al., 2017; Tarter et al., 2004), as well as elevated scores on a latent externalizing factor (Perkins et al., 2022). Third, factor analyses have often supported a highly heritable (Hicks et al., 2004; Krueger et al., 2002; Young et al., 2000) latent externalizing factor that includes ADHD along with other externalizing conditions (e.g., Krueger et al., 2005, 2018, 2021).

Still, other research supports a common cause explanation of ADHD's comorbidity with neurodevelopmental conditions (Andrews et al., 2009; Du Rietz et al., 2021; Michelini et al., 2019; Uhlhaas et al., 2023). As with other neurodevelopmental conditions, ADHD has a relatively early onset and persistent course relative to other psychiatric conditions (e.g., mood disorders), with symptoms emerging around preschool age and maintained into adolescence or adulthood (Nigg et al., 2020; Uhlhaas et al., 2023). Also, numerous studies have found evidence for shared genetic variation between ADHD and neurodevelopmental spectrum conditions (Andrews et al., 2009; Du Rietz et al., 2021; Glessner et al., 2017; Pettersson et al., 2023; Thapar et al., 2017), as well as shared neurobiological alterations (e.g., reduced white matter integrity, dorsolateral prefrontal cortex hypoactivation; Cortese et al., 2023; Hettwer et al., 2022; McCutcheon et al., 2023). At the phenotypic level, core features of ADHD (i.e., inattention, hyperactivity/impulsivity) are also common characteristics of other neurodevelopmental conditions (e.g., learning difficulties; Astle et al., 2019; Hawkins et al., 2016). Considering this body of literature, models that endorse the placement of ADHD under an externalizing spectrum may partly reflect the fact that their models either had limited or no coverage of neurodevelopmental spectrum conditions.

Indeed, a recent factor analysis supported the structural coherence of a neurodevelopmental spectrum that includes ADHD features alongside social problems and autism (Pettersson et al., 2023) when modeled together with externalizing factors (i.e., substance use, impulsivity). Another study that modeled ADHD as an individual factor found that, controlling for general psychopathology, ADHD had stronger phenotypic and genetic associations with the neurodevelopmental spectrum than the externalizing and internalizing spectra (Du Rietz et al., 2021).

### An alternative to the common cause model

The dominance of the common cause model of psychiatric comorbidity is closely tied to the classification literature's increasing reliance on factor analysis. Modern taxonomies typically use factor analysis, as opposed to alternative methods (e.g., latent class analysis, network analysis), to evaluate patterns of covariation among different forms of psychopathology and inform how putatively distinct conditions overlap and can be grouped into broad transdiagnostic dimensions. Technically, a latent factor only summarizes the shared variance among a set of indicators, although that shared variance is often taken to represent a causal latent variable, or an underlying construct. This interpretation dates back to the origins of factor analysis when Spearman (1904) developed the method to substantiate his theory of the general intelligence factor, which argued that correlations among different intelligence tests (e.g., verbal fluency, spatial ability) are caused by a single latent cognitive ability.

Nevertheless, more recent work has shown that positive correlations among cognitive abilities (e.g., working memory, information processing) that are traditionally attributed to a general intelligence factor can instead arise from a dynamic network that does not incorporate latent variables (Borsboom et al., 2011; Van Der Maas et al., 2006). Such a possibility aligns with a dynamic mutualism account of psychiatric comorbidity, whereby symptoms directly and positively interact and reinforce each other over time (Borsboom, 2017; Murray et al., 2016). These interactions may be unidirectional or bidirectional (van Bork et al., 2017). For example, insomnia may unidirectionally predict next‐day fatigue, whereas insomnia's relationship with anxiety may be bidirectional, such that insomnia increases anxiety, and anxiety in turn exacerbates insomnia. For ADHD, such a heterotypic reinforcement with other psychopathology may operate over a longer interval (e.g., 1 year), given its developmental stability and persistence in adolescence (Van Meter et al., 2024; Vergunst et al., 2019). Unlike anxiety and depression, which are largely anchored in negative affect (dysregulation) and are highly sensitive to proximal stressors (e.g., Lonigan et al., 2003; McLaughlin & Hatzenbuehler, 2009), ADHD symptom dimensions may exhibit greater temporal stability across development (e.g., Leopold et al., 2016, 2019; Vergunst et al., 2019).

Only a few studies have examined ADHD's comorbidity patterns in the context of a dynamic mutualism model, with most employing cross‐sectional network models that included ADHD symptoms in relation to either externalizing or neurodevelopmental features. Findings from these studies suggest that ADHD symptoms are closely connected with externalizing (Goulter et al., 2022; Karalunas et al., 2021; Liu et al., 2023; Martel et al., 2017; McElroy, Belsky, et al., 2018; Speyer et al., 2021) and not neurodevelopmental conditions or autism features in particular (Farhat et al., 2022). Given the limited literature, dynamic mutualism as an alternative explanation of ADHD's links with externalizing versus neurodevelopmental spectra remains elusive.

In a cross‐sectional study especially relevant to this investigation, Peng et al. (2025) used both factor analysis and network analysis to examine ADHD symptoms' associations with both externalizing and neurodevelopmental spectra using data from the Adolescent Brain Cognitive Development (ABCD) Study. Although ADHD symptoms showed differential associations with both spectra across different analytic frameworks, these findings cannot adjudicate between common cause and dynamic mutualism accounts of ADHD, as cross‐sectional models are inadequately equipped to inform psychopathology development and within‐person dynamics that result in comorbidity (Conlin et al., 2022; Fried & Cramer, 2017; McNally, 2021).

Longitudinal models that disaggregate within‐ and between‐person variation in psychopathology are best suited to track the development of ADHD's comorbidity over time. To date, longitudinal studies have only compared limited sets of developmental models (i.e., *p*‐differentiation, dynamic mutualism) in explaining the co‐occurrence of ADHD and internalizing or externalizing psychopathology (McElroy, Belsky, et al., 2018; Richards et al., 2024). No study has directly compared the common cause and dynamic mutualism models in explaining ADHD's comorbidity with externalizing versus neurodevelopmental spectra across development. Because psychopathology classification frameworks assume conditions within the same higher‐order domain as reflecting shared etiological liability (Beauchaine & McNulty, 2013; Krueger et al., 2005; Pettersson et al., 2013), evidence for the common cause model would support the placement of ADHD alongside another transdiagnostic spectrum and the importance of identifying transdiagnostic factors as targets for intervention. In contrast, support for the dynamic mutualism model would underscore the need to identify distinct, specific risk processes in the prevention of ADHD and co‐occurring psychopathology.

This exploratory study aimed to adjudicate between common cause and dynamic mutualism models in explaining the developmental links between ADHD and neurodevelopmental versus externalizing spectra to inform ADHD classification. To this end, we used data from a large cohort of youths (baseline *N* = 11,878, age = 9–10) who were followed across four waves and into early adolescence to examine ADHD's associations with externalizing and neurodevelopmental spectra. This developmental period is characterized by fluctuations of behaviors and symptoms, making it particularly informative for examining ADHD comorbidities.

## METHODS

### Data

We used data from the ABCD Study (release 5.0), which follows 11,878 children (ages 9–10; 48% female) from 21 sites in the United States. We used data from four available assessment waves: baseline (*n* = 11,861), the one‐year follow‐up (*n* = 11,213), the two‐year follow‐up (*n* = 10,966), and the three‐year follow‐up (*n* = 10,329). At baseline, youth self‐identified as 78% White, 10% Black, 2% Asian, and 10% Other, with 20% of identifying as Hispanic/Latino ethnicity. Combined household income was distributed as follows: $0–50,000 by 30%, $50,001–100,000 by 28%, and $100,001+ by 42%. Caregiver education was distributed as follows: 7% did not finish high school, 11% did, 29% attended some college, 28% earned a bachelor's degree, and 25% earned a graduate degree.

### Ethical Considerations

This secondary data analysis study did not require further ethical approval than that provided to the ABCD Study sites.

### Measures

#### Child Behavioral Checklist

We used symptoms from the Child Behavioral Checklist for Ages 6–18 (CBCL/6–18; 119 items; Achenbach et al., 2009) to characterize ADHD, the externalizing spectrum, and the neurodevelopmental spectrum (Supporting Information S2; Table S1). Symptoms were rated on a three‐point scale (*not true*, *somewhat true/sometimes true*, *very true/often true*).

**ADHD.** ADHD symptoms were selected from the Attention Problems subscale and the DSM‐oriented Attention Deficit/Hyperactivity scale (10 items, ωs = 0.92 across waves). Given ADHD's heterogeneity and evidence that its subcomponents relate differentially with externalizing and neurodevelopmental spectra (Martel et al., 2011; Michelini, et al., 2024; Peng et al., 2025), we examined ADHD at the global and subdimension level, based on prior work (Peng et al., 2025). We computed composites for ADHD and three subdimensions: inattention (e.g., “difficulty concentrating,” “inattentive”), hyperactivity/impulsivity (e.g., “difficulty sitting still,” “impulsive”), and cognitive disengagement (e.g., “confused,” “daydreamed”; Peng et al., 2025). Although a growing body of evidence supports cognitive disengagement as distinguishable from ADHD (e.g., Becker et al., 2016, 2020), we included cognitive disengagement symptoms to comprehensively capture attention difficulties in youth.

**Externalizing spectrum**. Externalizing symptoms were drawn from the Externalizing scale (30 items, ωs = 0.97 across waves). We removed five items that had extremely low base rates (i.e., “alcohol use,” “tobacco use,” “other drug use,” “vandalism,” “truant”; endorsed as “true” or “sometimes true” by < 2%) and one item that was included as an ADHD symptom (i.e., “loud”).

**Neurodevelopmental spectrum.** As in prior work (Ooi et al., 2011; So et al., 2013), the neurodevelopmental spectrum consists of 11 symptoms drawn from the Thought Problems, Attention Problems, Social Problems, and Internalizing scales from the CBCL (1 Attention Problems item, 3 Internalizing items, 6 Thought Problems items, 1 Social Problems, ωs = 0.89 across waves): acted young, restricted interests, peer difficulty, specific fears, preferred alone, twitching, motor clumsiness, repetitive behavior, language difficulty, atypical behavior, and social withdrawal. These items have been shown to effectively distinguish children with autism spectrum disorders from those with ADHD or internalizing disorders and capture varied developmental problems (Ooi et al., 2011; So et al., 2013), including social atypicality (e.g., “acted young”, “strange behavior”), motor dysfunction (e.g., “poor coordination,” “twitching”), and behavioral rigidity (e.g., “obsession,” “repetitive behaviors”).

Per modern effect size benchmarks (Funder & Ozer, 2019), neurodevelopmental spectrum scores showed very large correlations with scores from multiple CBCL subscales, including Aggressive Behavior (*r* = 0.59), Anxious/Depressed (*r* = 0.60), Attention Problems (*r* = 0.56), Rule‐Breaking Behavior (*r* = 0.50), Social Problems (*r* = 0.68), Somatic Complaints (*r* = 0.40), Thought Problems (*r* = 0.62), and Withdrawn/Depressed (*r* = 0.64). These findings suggest that the neurodevelopmental spectrum scale is not isomorphic with other CBCL subscales.

The ABCD Study also includes an additional scale that proxies neurodevelopmental spectrum features, the Short Social Responsiveness Scale (Sturm et al., 2017), at the 1‐year follow‐up, which prevents its use for the longitudinal approach adopted here. The CBCL Neurodevelopmental scale was strongly correlated with the SSRS at the 1‐year follow‐up (*r* = 0.74), suggesting strong convergent validity of our neurodevelopmental scale (Supporting Information S2; Table S2).

### Analyses

Prior to model estimation, we computed intraclass correlation coefficients (ICCs) for ADHD and psychopathology spectra to verify sufficient between‐ and within‐person variability in ADHD and other psychopathology (Hoffman & Stawski, 2009; Koo & Li, 2016) using the performance package in *R* version 4.4.1 (R Core Team, 2023).

To model the co‐development of ADHD and other psychopathology over time, we used Mplus version 8.5 (Muthén & Muthén, 2020) to estimate a latent curve model with structured residuals (LCM‐SR; Curran et al., 2014), which partitions between‐ and within‐person sources of variance while also modeling developmental trajectories, making it unique among its competitors (Curran et al., 2014; Orth et al., 2021).^1^ We used the robust maximum likelihood (MLR) estimator and accounted for the ABCD Study's complex survey design by treating study site as a stratum variable and family ID as a cluster variable. Model fit was evaluated using established guidelines: comparative fit index (CFI) ≥ 0.95, root mean square error of approximation (RMSEA) ≤ 0.5, and a standardized root mean square residual (SRMR) ≤ 0.05.^2^

**FIGURE 1:**
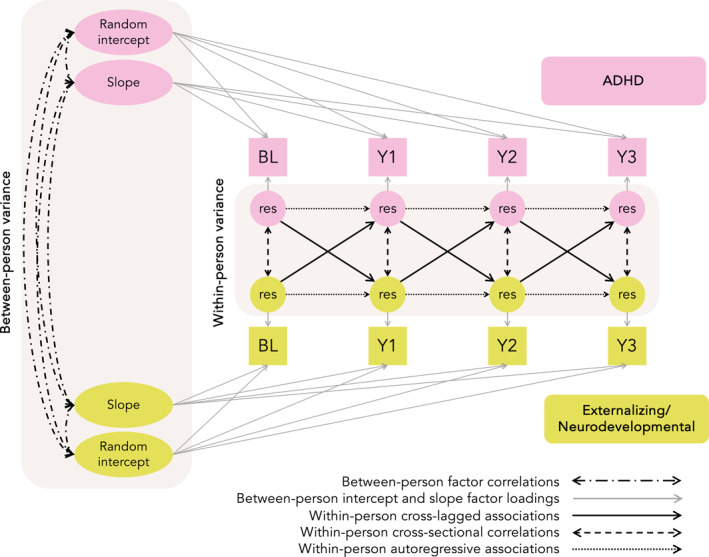
An illustration of the latent curve model with structured residuals. BL = baseline; Y1 = 1‐year follow‐up; Y2 = 2‐year follow‐up; Y3 = 3‐year follow‐up.

The LCM‐SR contains five kinds of parameters (Figure 1). First, the LCM‐SR includes a random intercept for each construct (i.e., ADHD, externalizing) that effectively reflects a given participant's mean level of each construct over time (i.e., their trait level). In short, the random intercepts account for the possibility that participants have different starting points in terms of their level of a given construct at baseline. Second, the LCM‐SR includes a random slope for each construct. Whereas the random intercept factor captures a person's mean on each construct, the slope factor captures a person's specific trajectory of the construct over time, or their rate of change in the construct over time.

Third, the LCM‐SR includes autoregressive associations between adjacent waves of each construct with itself over time (e.g., ADHD at baseline → ADHD at the 1‐year follow‐up). Fourth, the LCM‐SR includes cross‐lagged paths that capture the prospective associations between one construct at one wave and another at the subsequent wave (e.g., ADHD at baseline → externalizing at the 1‐year follow‐up). In the LCM‐SR, the cross‐lagged associations reflect whether a within‐person deviation from the trait level of one construct has a prospective relationship with change in the within‐person deviation from the trait level of the other construct. Fifth, the LCM‐SR includes within‐person cross‐sectional correlations among both sets of constructs across all waves. They indicate how two constructs are associated with each other at a given time point after accounting for person‐specific deviations from their average levels and deviations from their growth trajectories while accounting for autoregressive associations.

**FIGURE 2:**
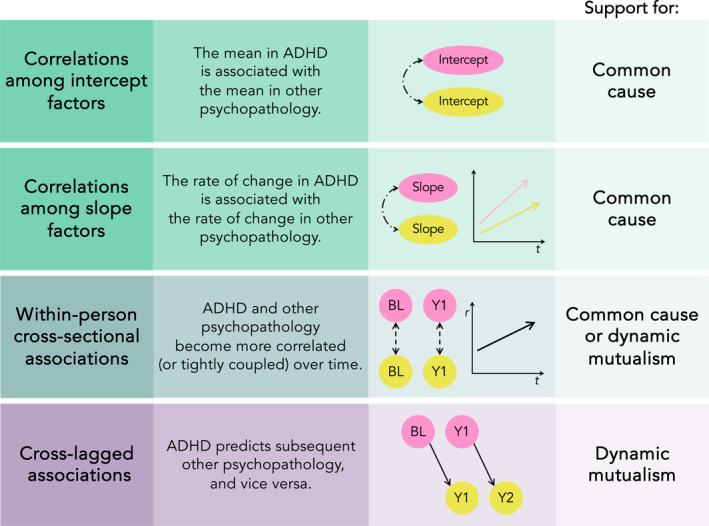
Key parameters from the latent curve model with structured residuals that map onto common cause and dynamic mutualism models. BL = baseline; Y1 = 1‐year follow‐up; Y2 = 2‐year follow‐up; Y3 = 3‐year follow‐up.

Evidence supporting the common cause model includes substantial correlations among the random intercepts and slopes for ADHD and another psychopathology (Figure 2). This pattern suggests that ADHD and other psychopathology are stably coupled across development, both in their average severity and trajectories of change, expected under a shared underlying liability. In contrast, evidence supporting the dynamic mutualism model includes substantial cross‐lagged associations whereby ADHD predicts later psychopathology or vice versa, as dynamic mutualism predicts longitudinal heterotypic associations. Finally, large within‐person cross‐sectional associations across waves are necessary but not sufficient to support both the common cause and dynamic mutualism models, as both frameworks predict psychopathology concurrent overlap within persons. We interpreted the magnitude of the standardized parameter estimates (*B*s and *r*s) using Funder and Ozer's (2019) benchmarks: 0.05 = very small, 0.10 = small, 0.20 = medium, 0.30 = large, and 0.40 = very large.

## RESULTS

### Between‐ and within‐person variability

Across the four waves of data, 72% of the variance in ADHD was attributable to between‐person variability (i.e., ICC = 0.72), with the remaining 28% being attributable to within‐person variability. Between‐person variability was similarly large for ADHD subdimensions, at 67% for inattention, 68% for hyperactivity, and 57% for cognitive disengagement. Likewise, 65% of the variance in both externalizing and neurodevelopmental spectra was attributable to between‐person variance, with 35% attributable to within‐person variance (Supporting Information S2; Table S5).

### Model fit

Both sets of LCM‐SRs, one for the externalizing spectrum and one for the neurodevelopmental spectrum, fit well according to conventional benchmarks (Supporting Information S2; Table S6; externalizing and global ADHD: CFI = 0.999, RMSEA = 0.02, SRMR = 0.01; neurodevelopmental and global ADHD: CFI = 0.997, RMSEA = 0.04, SRMR = 0.01). Supporting Information S2; Table S7 contains all model parameters.

### Evidence for common cause model

#### Externalizing spectrum

The random intercepts for ADHD and the externalizing spectrum were strongly correlated (*r* = 0.66), as were the random slopes (*r* = 0.46). These findings held for ADHD subdimensions, although the strength of the association varied as a function of subdimension.

**FIGURE 3:**
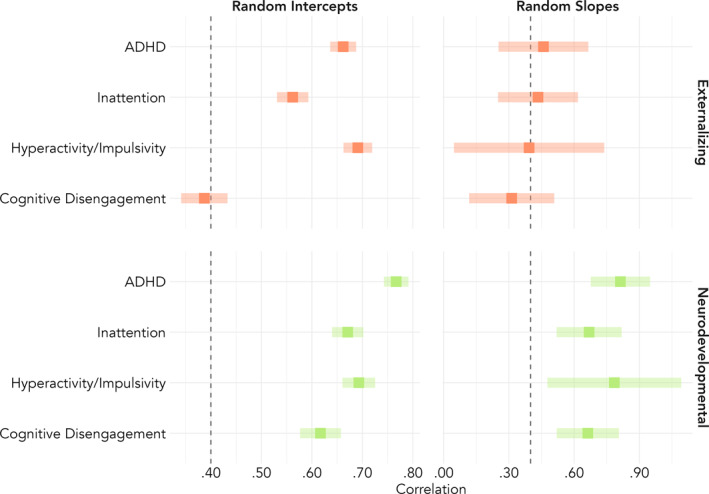
Correlations between random intercepts and slopes for ADHD and externalizing and neurodevelopmental spectra. The squares reflect correlation coefficients, and the shaded area around them reflects the 83% confidence interval. We used an 83% rather than a 95% confidence interval to avoid overly stringent comparisons of estimates. The dashed gray line reflects a very large correlation (*r* = 0.40), based on benchmarks defined by Funder and Ozer (2019). ADHD = attention‐deficit/hyperactivity disorder.

Correlations between random intercepts for ADHD subdimensions and the externalizing spectrum (range of *r*s: 0.39 to 0.69) ranged from large for cognitive disengagement (*r* = 0.39) to very large for inattention (*r* = 0.56) and hyperactivity/impulsivity (*r* = 0.69; Table 1, Figure 3). Correlations between random slopes for ADHD subdimensions and the externalizing spectrum (range: 0.31 to 0.43) also ranged from large to very large: cognitive disengagement (*r* = 0.31), hyperactivity/impulsivity (*r* = 0.39), and inattention (*r* = 0.43).

**TABLE 1:** Key parameters from the latent curve model with structured residuals that bear on the common cause model.

	Externalizing		Neurodevelopmental	
	*B*	SE	*B*	SE
Correlation between random intercepts				
ADHD	0.66	0.02	0.77	0.02
Inattention	0.56	0.02	0.67	0.02
Hyperactivity/Impulsivity	0.69	0.02	0.69	0.02
Cognitive disengagement	0.39	0.03	0.62	0.03
Correlation between random slopes				
ADHD	0.46	0.15	0.81	0.10
Inattention	0.43	0.14	0.67	0.11
Hyperactivity/Impulsivity	0.39	0.26	0.79	0.23
Cognitive disengagement	0.31	0.15	0.66	0.11
Within‐person cross‐sectional associations				
ADHD				
Baseline	0.53	0.05	0.44	0.06
One‐year follow‐up	0.48	0.02	0.46	0.02
Two‐year follow‐up	0.52	0.03	0.39	0.04
Three‐year follow‐up	0.57	0.07	0.27	0.15
Inattention				
Baseline	0.32	0.07	0.25	0.06
One‐year follow‐up	0.30	0.03	0.33	0.02
Two‐year follow‐up	0.35	0.03	0.25	0.04
Three‐year follow‐up	0.36	0.06	0.18	0.11
Hyperactivity/Impulsivity				
Baseline	0.55	0.05	0.38	0.05
One‐year follow‐up	0.47	0.03	0.37	0.02
Two‐year follow‐up	0.46	0.03	0.33	0.04
Three‐year follow‐up	0.51	0.06	0.23	0.12
Cognitive disengagement				
Baseline	0.27	0.07	0.34	0.06
One‐year follow‐up	0.30	0.03	0.37	0.03
Two‐year follow‐up	0.27	0.04	0.22	0.05
Three‐year follow‐up	0.27	0.09	0.06	0.20

*Note*: All parameters (e.g., the correlation between the random intercepts) are between the ADHD variable (row) and a psychopathology spectrum (column). Supporting Information S2; Table S7 contains all parameters.

Abbreviation: ADHD = attention‐deficit/hyperactivity disorder.

#### Neurodevelopmental spectrum

The random intercepts for ADHD and the neurodevelopmental spectrum were very strongly correlated (*r* = 0.77), as were the random slopes (*r* = 0.81). Similarly, correlations between random intercepts for ADHD subdimensions and the neurodevelopmental spectrum were all very large (*r* = 0.62 [cognitive disengagement], *r* = 0.67 [inattention], *r* = 0.69 [hyperactivity/impulsivity]; Table 1, Figure 3), as were the correlations between the random slopes (*r* = 0.66 [cognitive disengagement], *r* = 0.67 [inattention], *r* = 0.79 [hyperactivity/impulsivity]).

### Evidence for either common cause or dynamic mutualism

#### Externalizing spectrum

**FIGURE 4:**
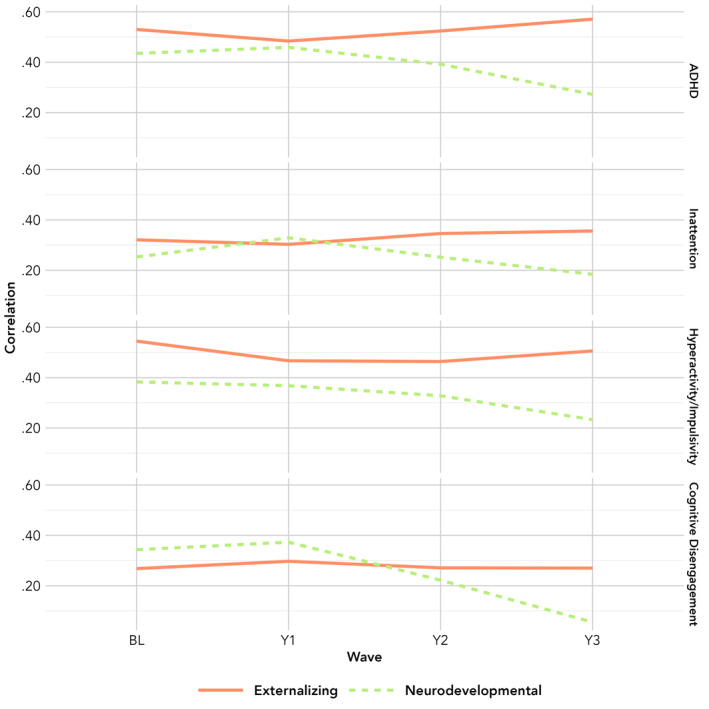
Within‐person cross‐sectional associations between ADHD and externalizing and neurodevelopmental spectra. ADHD = attention‐deficit/hyperactivity disorder; BL = baseline; Y1 = 1‐year follow‐up; Y2 = 2‐year follow‐up; Y3 = 3‐year follow‐up.

Within‐person cross‐sectional associations between ADHD and externalizing were stable across time (range of *B*s: 0.53 at baseline to 0.57 at 3‐year follow‐up; Table 1, Figure 4). Inattention (range: 0.30 to 0.36) and hyperactivity/impulsivity (range: 0.46 to 0.55) had large within‐person cross‐sectional associations with externalizing, whereas cognitive disengagement had medium associations (range: 0.27 to 0.30).

#### Neurodevelopmental spectrum

Within‐person cross‐sectional associations between ADHD and the neurodevelopmental spectrum were medium to very large, and they decreased over time (range of *B*s: 0.46 at baseline to 0.27 at 3‐year follow‐up; Table 1; Figure 4). These findings held for ADHD subdimensions (ranges [baseline to 3‐year follow‐up]: 0.33 to 0.18 [inattention]; 0.38 to 0.23 [hyperactivity/impulsivity]; 0.37 to 0.06 [cognitive disengagement]).

### Evidence for dynamic mutualism

#### Externalizing spectrum

Cross‐lagged associations whereby earlier ADHD prospectively predicted the externalizing spectrum (range of *B*s: 0.00–0.08) and vice versa (range: −0.02 to 0.11) were all very small to small (Table 2; Supporting Information S2; Figure S1). These findings held across ADHD subdimensions (ranges: −0.01 to 0.05 [inattention]; 0.00–0.11 [hyperactivity/impulsivity]; 0.00–0.04 [cognitive disengagement]).

**TABLE 2:** Key parameters from the latent curve model with structured residuals that bear on the dynamic mutualism model.

	ADHD		Inattention		Hyperactivity/Impulsivity		Cognitive disengagement	
	*B*	SE	*B*	SE	*B*	SE	*B*	SE
Cross‐lagged associations								
Externalizing								
BL ADHD → Y1 externalizing	0.08	0.06	0.00	0.04	0.11	0.06	0.04	0.05
Y1 ADHD → Y2 externalizing	0.00	0.03	−0.01	0.02	0.00	0.03	0.00	0.03
Y2 ADHD → Y3 externalizing	0.08	0.07	0.05	0.05	0.08	0.06	0.01	0.06
BL externalizing → Y1 ADHD	−0.02	0.05	0.00	0.05	0.03	0.05	0.02	0.05
Y1 externalizing → Y2 ADHD	0.04	0.03	0.04	0.02	0.03	0.03	0.04	0.02
Y2 externalizing → Y3 ADHD	0.11	0.08	0.03	0.06	0.05	0.07	0.03	0.07
Neurodevelopmental								
BL ADHD → Y1 neurodevelopmental	0.07	0.04	0.02	0.03	0.07	0.03	0.05	0.04
Y1 ADHD → Y2 neurodevelopmental	0.03	0.02	0.02	0.02	0.02	0.02	0.03	0.02
Y2 ADHD → Y3 neurodevelopmental	−0.12	0.10	−0.04	0.07	−0.03	0.08	−0.20	0.11
BL neurodevelopmental → Y1 ADHD	0.01	0.04	0.01	0.04	0.03	0.04	0.09	0.05
Y1 neurodevelopmental → Y2 ADHD	0.00	0.02	0.01	0.02	0.02	0.02	−0.01	0.03
Y2 neurodevelopmental → Y3 ADHD	−0.08	0.08	−0.05	0.06	−0.06	0.07	−0.16	0.09

*Note*: All parameters are between the ADHD variable (column) and a psychopathology spectrum (row). Supporting Information S2; Table S7 contains all parameters.

Abbreviations: ADHD = attention‐deficit/hyperactivity disorder; BL = baseline; Y1 = 1‐year follow‐up; Y2 = 2‐year follow‐up; Y3 = 3‐year follow‐up.

#### Neurodevelopmental spectrum

Cross‐lagged associations whereby earlier ADHD prospectively predicted the neurodevelopmental spectrum (range of *B*s: −0.12 to 0.07) and vice versa (range: −0.08 to 0.01) were all very small to small (Table 2; Supporting Information S2; Figure S1). These findings held across ADHD subdimensions (ranges: −0.05 to 0.02 [inattention]; −0.06 to 0.07 [hyperactivity/impulsivity]; −0.20 to 0.09 [cognitive disengagement]).

## DISCUSSION

Modern psychopathology classification systems differ in their placement of ADHD, either as a neurodevelopmental or an externalizing condition, with empirical evidence supporting both decisions (Michelini et al., 2024; Peng et al., 2025). Regardless of where ADHD is positioned in the psychopathology hierarchy, both approaches conceptualize ADHD's comorbidity with either neurodevelopmental or externalizing conditions as the product of the common cause model, whereby a shared set of influences gives rise to conditions under each spectrum (e.g., Kendler et al., 2003; Krueger, 1999a, 1999b; Krueger & Markon, 2006). Emerging conceptualizations, such as dynamic mutualism, posit that comorbidity might arise through alternative processes (Borsboom, 2017; Cramer et al., 2010a; Van Der Maas et al., 2006).

Our findings favored the common cause model over dynamic mutualism in explaining ADHD's ties to the externalizing and neurodevelopmental spectra across development. At the between‐person level, there was strong overlap between ADHD and both the externalizing and neurodevelopmental spectra. At the within‐person level, contemporaneous cross‐sectional associations between ADHD and the externalizing spectrum were moderate to strong and stable as opposed to increasing over time (Beauchaine et al., 2017). In contrast, within‐person cross‐sectional associations between ADHD and the neurodevelopmental spectrum were moderate and decreased steadily over time, suggesting differentiation across development.

Regarding externalizing, our findings partially corroborate Beauchaine and colleagues' (2017) theoretical framework of the externalizing spectrum, wherein ADHD emerges as an early form of externalizing: Initial levels of ADHD and externalizing were strongly correlated, as were their developmental trajectories. Nevertheless, this model posits that ADHD should become more closely linked with externalizing across development, and we did not find strong evidence for this supposition. Of ADHD subdimensions, hyperactivity/impulsivity had the strongest association with the externalizing spectrum, consistent with Beauchaine and colleagues' (2017) assertion that impulsivity is the underlying liability to all forms of externalizing psychopathology.

The phenotypic and etiologic link between ADHD and the externalizing spectrum is well supported. Specifically, numerous studies find a coherent latent externalizing factor that includes varied combinations of phenomena associated with poor behavioral control (e.g., conduct disorder, oppositional defiant disorder; Krueger et al., 2005, 2005, 2018, 2021) that appears to capture shared genetic influences (e.g., Capusan et al., 2015; Chang et al., 2012; Nadder et al., 2002; Ribasés et al., 2023). As evidenced here, such coherence between ADHD and the externalizing spectrum might be particularly strong for hyperactivity/impulsivity, with some evidence suggesting that hyperactivity/impulsivity and not inattention prospectively predict externalizing behaviors (e.g., alcohol dependence; Ahmad & Hinshaw, 2017; Edwards & Kendler, 2012).

Even though Beauchaine and colleagues' model of ADHD's comorbidity with externalizing appears reasonably well supported in our study, it is worth noting that we also observed evidence for the common cause model in explaining ADHD's comorbidity with the neurodevelopmental spectrum. Notably, although impulsivity/hyperactivity is considered a core externalizing feature, it also formed strong developmental ties with the neurodevelopmental spectrum, consistent with evidence that impulsivity is captured by both externalizing and neurodevelopmental dimensions (Holmes et al., 2021). Were Beauchaine and colleagues' model to hold entirely, we should see stronger links between ADHD dimensions and the externalizing spectrum than we did for the neurodevelopmental spectrum.

In fact, we observed even stronger evidence for the common cause model in explaining ADHD's comorbidity with neurodevelopmental and externalizing spectra, with evidence being significantly stronger for the former when considering the magnitude of associations between the random intercepts, in particular. Of ADHD subdimensions, inattention had a stronger between‐person association with the neurodevelopmental spectrum than the externalizing spectrum. This pattern aligns with previous factor analytic studies, which consistently yielded a (neurodevelopmental) dimension that captures both inattention and ASD features, with inattention showing negligible loadings on the externalizing dimension (Afzali et al., 2018; Carragher et al., 2014, 2016; Laceulle et al., 2015; Martel et al., 2017; Michelini et al., 2019).

Likewise, cognitive disengagement had a stronger between‐person association with the neurodevelopmental spectrum than did other ADHD dimensions. Such strong associations were potentially driven by a subset of neurodevelopmental items related to low social engagement (e.g., “withdrawn,” “preferred being alone”; Ooi et al., 2011; So et al., 2013). Prior work has shown that cognitive disengagement contributes to increased withdrawal and isolation beyond ADHD or internalizing problems (Becker et al., 2019, 2024; Rondon et al., 2020; Servera et al., 2018), motivating future research to ascertain the mechanisms underlying the strong associations between cognitive disengagement and the neurodevelopmental spectrum. With that said, the notion that cognitive disengagement might beget social isolation did not bear out in the cross‐lagged associations that might support relations posited by dynamic mutualism.

The stronger connection between ADHD dimensions and the neurodevelopmental spectrum than the externalizing spectrum is consistent with findings from genetic studies. Du Rietz et al. (2021), for instance, demonstrated that ADHD's association with the neurodevelopmental spectrum was moderately strong and largely influenced by shared genetic effects, whereas its links to the externalizing factor were weaker and driven primarily by nonshared environmental influences. Recent molecular genetic studies also found that ADHD polygenic risk scores were uniquely associated with the neurodevelopmental spectrum and not the externalizing spectrum (Waszczuk et al., 2023).

In contrast with the stable within‐person cross‐sectional associations between ADHD and the externalizing spectrum across development, we observed decreasing associations between ADHD and the neurodevelopmental spectrum. These findings imply that ADHD and neurodevelopmental spectrum phenomena become more differentiable over time, which may reflect a natural process of psychopathology differentiation, during which a broader psychopathology liability unfolds into increasingly specific forms of psychopathology over time (e.g., Lilienfeld et al., 1994; Patterson, 1995; Sterba et al., 2010). One potential explanation may be the development or maturation of higher‐order cognitive abilities (e.g., metacognitive capacity, verbal ability) that typically emerge later in development, particularly during late childhood to early adolescence (Van der Stel & Veenman, 2010; Weil et al., 2013). As these capabilities develop, parents may become better able to differentiate ADHD from neurodevelopmental conditions, leading to increasingly divergent reports. For instance, a nonverbal child may manifest with behavioral problems, which may be taken to indicate both ADHD and autism, but as verbal and higher‐level social abilities begin to develop, it may help distinguish the child's autism from ADHD.

A related explanation for the differentiation between ADHD and the neurodevelopmental spectrum is the “generalist genes, specialist environments” hypothesis, whereby environmental influences (e.g., social context, coping strategies) guide shared genetic liability across ADHD and neurodevelopmental conditions to domain‐specific manifestations over time (Eley, 1997; Kovas & Plomin, 2007). For instance, a subset of children who exhibit symptoms of ADHD may experience social rejection from peers and differential treatment from teachers, nudging them toward displaying externalizing behaviors (e.g., aggression, defiance; Evans et al., 2015; Mrug et al., 2012; Obsuth et al., 2020) rather than neurodevelopmental features (e.g., working memory issues, motor issues).

Taken together, our findings favor the common cause model over dynamic mutualism in explaining ADHD's developmental links to the externalizing and the neurodevelopmental spectra, aligning with available longitudinal studies showing little evidence that changes in ADHD drive changes in externalizing or neurodevelopmental symptoms, or vice versa (Farhat et al., 2022; McElroy, Belsky, et al., 2018). In studies that found support for dynamic mutualism, researchers commonly rely on cross‐sectional network analysis that fails to capture longitudinal relations or parse within‐ and between‐person effects (e.g., McElroy, Shevlin, et al., 2018; Silk et al., 2019; Speyer et al., 2021). Thus, our longitudinal approach likely captures more accurate developmental relationships between ADHD and its comorbidities.

### Limitations, factors that complicate theory testing, and future directions

Interpretation of our findings warrants discussion of several important factors. First, common method variance (Campbell & Fiske, 1959; De Los Reyes et al., 2015) may have inflated correlations among the random intercepts of ADHD and other psychopathology spectra, exaggerating support for the common cause model. Method biases, such as evaluative consistency bias—the tendency to consistently appraise oneself or others in overly positive or negative ways across traits—could systematically rule out caregiver reports (Feeley, 2002; Forgas & Laham, 2016). For example, caregivers with negative biases may misattribute normative behaviors (e.g., spirited, assertive) to pathology (e.g., hyperactive, defiant), artificially increasing the overlap between ADHD and other forms of psychopathology.

Second, although we did not find evidence for dynamic mutualism in explaining the developmental ties between ADHD and other forms of psychopathology, since we found no cross‐lagged associations, such lack of support does not entirely rule out a dynamic mutualism account of ADHD's comorbidity. Instead, the absence of substantial cross‐lagged associations may reflect a mismatch between the causal process and the assessment intervals in our sample (Cole, 2006; Dormann & Griffin, 2015; Griep et al., 2021). ADHD may predict other forms of psychopathology over distinct timeframes that differ from the 1‐year intervals captured in the ABCD study (e.g., months, years). For instance, prior research has found that ADHD symptoms predict externalizing behaviors over approximately 3 years (Ralf et al., 2015). Given substantial developmental fluctuations in mental health during early adolescence and the temporal stability of ADHD symptom presentations, as opposed to transient or context‐specific variation (Dalsgaard et al., 2020), a 6‐month interval may optimally balance symptom co‐variation with developmental change. Ultimately, support for dynamic mutualism will remain elusive until theory informs more precise time‐bound relationships between ADHD and other forms of psychopathology (Aristodemou et al., 2024; Fried, 2020; Robinaugh et al., 2021).

Finally, our assessment of the neurodevelopmental spectrum relied on CBCL symptoms. Although previous research suggests that items such as “social withdrawal” and “social oddity” can distinguish autism from ADHD or internalizing disorders (Hoffmann et al., 2016; Ooi et al., 2011; So et al., 2013), measures that capture a broader range of neurodevelopmental conditions (e.g., Tourette's disorder, motor disorders) may be necessary to fully articulate ADHD's relationship with the neurodevelopmental spectrum. Relatedly, future work could extend longitudinal models to integrate other externalizing conditions, such as substance use disorders that emerge later in development.

## CONCLUSION

Our study contributes to the ongoing discourse surrounding whether ADHD should be classified as a neurodevelopmental or externalizing disorder in contemporary classification systems, with findings supporting positioning ADHD under both the neurodevelopmental and externalizing spectra and the common cause model in both cases. Of ADHD subdimensions, cognitive disengagement had the strongest between‐person ties to the neurodevelopmental spectrum, whereas hyperactivity/impulsivity had the strongest ties to the externalizing spectrum. Together, our findings suggest that ADHD should not reside exclusively within either spectrum, and that the common cause model was favored over dynamic mutualism in these data.

## AUTHOR CONTRIBUTIONS

**Zheyue Peng**: Conceptualization; investigation; writing—original draft; methodology; visualization; writing—review and editing; formal analysis; data curation. **Ashley L. Watts**: Conceptualization; investigation; funding acquisition; methodology; visualization; writing—original draft; writing—review and editing; supervision.

## CONFLICT OF INTEREST STATEMENT

The authors declare no conflicts of interest.

## ETHICAL CONSIDERATIONS

Most ABCD research sites rely on a central Institutional Review Board (IRB) at the University of California, San Diego, for the ethical review and approval of the research protocol, with a few sites obtaining local IRB approval.

## Supporting information

Supporting Information S1

Supporting Information S2

## Data Availability

The data that support the findings of this study are openly available in NIH Brain Development Cohorts (NBDC) Data Hub at https://www.nbdc‐datahub.org, reference number 10.15154/8873‐zj65.

## ENDNOTES

^1^
We also considered the random intercept cross‐lagged panel model and the autoregressive latent trajectory model. The autoregressive latent trajectory model models did not converge, and results for the random intercept cross‐lagged panel model are presented in Supporting Information S2; Table S3. Results from the random intercept cross‐lagged panel model were consistent with those presented here.

^2^
We also estimated all primary models separately for each sex, whereby boys and girls are specified as separate groups (Supporting Information S1; Appendix 1 and Supporting Information S2; Table S4). Results did not differ dramatically across sex, with some exceptions. First, the random intercepts for ADHD and its subdimensions (i.e., inattention, hyperactivity/impulsivity) were more strongly associated with both the externalizing and neurodevelopmental spectra in boys than in girls (Peng et al., in press). Second, the random intercepts and slopes for ADHD were more strongly associated with those for the neurodevelopmental spectrum than the externalizing spectrum in boys, whereas this pattern was not observed in girls. In fact, the random intercept for ADHD was more strongly associated with the externalizing spectrum than the neurodevelopmental spectrum in girls.

## References

[jcv270142-bib-0001] Achenbach, T. (2009). Achenbach system of empirically based assessment (ASEBA): Development, findings, theory, and applications. Encyclopedia of Autism Spectrum Disorders.

[jcv270142-bib-0002] Afzali, M. H. , Sunderland, M. , Carragher, N. , & Conrod, P. (2018). The Structure of Psychopathology in Early Adolescence: Study of a Canadian Sample: La structure de la psychopathologie au début de l’adolescence: étude d’un échantillon canadien. Canadian Journal of Psychiatry, 63(4), 223–230. 10.1177/0706743717737032 29061067 PMC5894914

[jcv270142-bib-0003] Ahmad, S. I. , & Hinshaw, S. P. (2017). Attention‐Deficit/Hyperactivity disorder, trait impulsivity, and externalizing behavior in a longitudinal sample. Journal of Abnormal Child Psychology, 45(6), 1077–1089. 10.1007/s10802-016-0226-9 27838891 PMC5429206

[jcv270142-bib-0004] American Psychiatric Association . (2013). In Diagnostic and statistical manual of mental disorders: DSM‐5 (5th ed.). American Psychiatric Association. 10.1176/appi.books.9780890425596

[jcv270142-bib-0005] Andrews, G. , Pine, D. S. , Hobbs, M. J. , Anderson, T. M. , & Sunderland, M. (2009). Neurodevelopmental disorders: Cluster 2 of the proposed meta‐structure for DSM‐V and ICD‐11: Paper 3 of 7 of the thematic section: ‘A proposal for a meta‐structure for DSM‐V and ICD‐11’. Psychological Medicine, 39(12), 2013–2023. 10.1017/S0033291709990274 19796427 PMC3006670

[jcv270142-bib-0006] Angold, A. , Costello, E. J. , & Erkanli, A. (1999). Comorbidity. Journal of Child Psychology and Psychiatry, 40(1), 57–87. 10.1111/1469-7610.00424 10102726

[jcv270142-bib-0007] Aristodemou, M. E. , Kievit, R. A. , Murray, A. L. , Eisner, M. , Ribeaud, D. , & Fried, E. I. (2024). Common cause versus dynamic mutualism: An empirical comparison of two theories of psychopathology in two large longitudinal cohorts. Clinical Psychological Science, 12(3), 380–402. 10.1177/21677026231162814 38827924 PMC11136614

[jcv270142-bib-0008] Astle, D. E. , Bathelt, J. , Calm Team , & Holmes, J. (2019). Remapping the cognitive and neural profiles of children who struggle at school. Developmental Science, 22(1), e12747. 10.1111/desc.12747 30171790 PMC6808180

[jcv270142-bib-0009] Beauchaine, T. P. , Hinshaw, S. P. , & Pang, K. L. (2010). Comorbidity of attention‐deficit/hyperactivity disorder and early‐onset conduct disorder: Biological, environmental, and developmental mechanisms. Clinical Psychology: Science and Practice, 17(4), 327Â–336. 10.1111/j.1468-2850.2010.01224.x

[jcv270142-bib-0010] Beauchaine, T. P. , & McNulty, T. (2013). Comorbidities and continuities as ontogenic processes: Toward a developmental spectrum model of externalizing psychopathology. Development and Psychopathology, 25(4), 1505–1528. 10.1017/S0954579413000746 24342853 PMC4008972

[jcv270142-bib-0011] Beauchaine, T. P. , Zisner, A. R. , & Sauder, C. L. (2017). Trait impulsivity and the externalizing spectrum. Annual Review of Clinical Psychology, 13(1), 343–368. 10.1146/annurev-clinpsy-021815-093253 28375718

[jcv270142-bib-0012] Becker, S. P. , Burns, G. L. , Smith, Z. R. , & Langberg, J. M. (2020). Sluggish cognitive tempo in adolescents with and without ADHD: Differentiation from adolescent‐reported ADHD inattention and unique associations with internalizing domains. Journal of Abnormal Child Psychology, 48(3), 391–406. 10.1007/s10802-019-00603-9 31814060 PMC7007365

[jcv270142-bib-0013] Becker, S. P. , Garner, A. A. , Tamm, L. , Antonini, T. N. , & Epstein, J. N. (2019). Honing in on the social difficulties associated with sluggish cognitive tempo in children: Withdrawal, peer ignoring, and low engagement. Journal of Clinical Child and Adolescent Psychology, 48(2), 228–237. 10.1080/15374416.2017.1286595 28287826 PMC6047915

[jcv270142-bib-0014] Becker, S. P. , Leopold, D. R. , Burns, G. L. , Jarrett, M. A. , Langberg, J. M. , Marshall, S. A. , McBurnett, K. , Waschbusch, D. A. , & Willcutt, E. G. (2016). The internal, external, and diagnostic validity of sluggish cognitive tempo: A meta‐analysis and critical review. Journal of the American Academy of Child & Adolescent Psychiatry, 55(3), 163–178. 10.1016/j.jaac.2015.12.006 26903250 PMC4764798

[jcv270142-bib-0015] Becker, S. P. , Vaughn, A. J. , Zoromski, A. K. , Burns, G. , Leonard, M. , Amori, Y. , Fredrick, J. W. , Epstein, J. N. , Peugh, J. L. , & Tamm, L. (2024). A multi‐method examination of peer functioning in children with and without cognitive disengagement syndrome. Journal of Clinical Child and Adolescent Psychology, 1–16. 10.1080/15374416.2024.2301771 PMC11231062 38193746

[jcv270142-bib-0016] Borsboom, D. (2017). A network theory of mental disorders. World Psychiatry, 16(1), 5–13. 10.1002/wps.20375 28127906 PMC5269502

[jcv270142-bib-0017] Borsboom, D. , Cramer, A. O. , Schmittmann, V. D. , Epskamp, S. , & Waldorp, L. J. (2011). The small world of psychopathology. PLoS One, 6(11), e27407. 10.1371/journal.pone.0027407 22114671 PMC3219664

[jcv270142-bib-0018] Burke, J. D. , Loeber, R. , & Lahey, B. B. (2001). Which aspects of ADHD are associated with tobacco use in early adolescence? The Journal of Child Psychology and Psychiatry and Allied Disciplines, 42(4), 493–502. 10.1111/1469-7610.00743 11383965

[jcv270142-bib-0019] Burt, S. A. , Krueger, R. F. , McGue, M. , & Iacono, W. G. (2001). Sources of covariation among attention‐deficit/hyperactivity disorder, oppositional defiant disorder, and conduct disorder: The importance of shared environment. Journal of Abnormal Psychology, 110(4), 516–525. 10.1037/0021-843x.110.4.516 11727941

[jcv270142-bib-0020] Campbell, D. T. , & Fiske, D. W. (1959). Convergent and discriminant validation by the multitrait‐multimethod matrix. Psychological Bulletin, 56(2), 81–105. 10.1037/h0046016 13634291

[jcv270142-bib-0021] Capusan, A. J. , Bendtsen, P. , Marteinsdottir, I. , Kuja‐Halkola, R. , & Larsson, H. (2015). Genetic and environmental contributions to the association between attention deficit hyperactivity disorder and alcohol dependence in adulthood: A large population‐based twin study. American Journal of Medical Genetics Part B: Neuropsychiatric Genetics, 168(6), 414–422. 10.1002/ajmg.b.32300 25711682

[jcv270142-bib-0022] Capusan, A. J. , Bendtsen, P. , Marteinsdottir, I. , & Larsson, H. (2019). Comorbidity of adult ADHD and its subtypes with substance use disorder in a large population‐based epidemiological study. Journal of Attention Disorders, 23(12), 1416–1426. 10.1177/1087054715626511 26838558

[jcv270142-bib-0023] Carragher, N. , Krueger, R. F. , Eaton, N. R. , Markon, K. E. , Keyes, K. M. , Blanco, C. , Saha, T. D. , & Hasin, D. S. (2014). ADHD and the externalizing spectrum: Direct comparison of categorical, continuous, and hybrid models of liability in a nationally representative sample. Social Psychiatry and Psychiatric Epidemiology, 49(8), 1307–1317. 10.1007/s00127-013-0770-3 24081325 PMC3972373

[jcv270142-bib-0025] Chang, Z. , Lichtenstein, P. , & Larsson, H. (2012). The effects of childhood ADHD symptoms on early‐onset substance use: A Swedish twin study. Journal of Abnormal Child Psychology, 40(3), 425–435. 10.1007/s10802-011-9575-6 21947618

[jcv270142-bib-0026] Cole, D. A. (2006). Coping with longitudinal data in research on developmental psychopathology. International Journal of Behavioral Development, 30(1), 20–25. 10.1177/0165025406059969

[jcv270142-bib-0027] Conlin, W. E. , Hoffman, M. , Steinley, D. , & Sher, K. J. (2022). Cross‐sectional and longitudinal AUD symptom networks: They tell different stories. Addictive Behaviors, 131, 107333. 10.1016/j.addbeh.2022.107333 35429920 PMC9491298

[jcv270142-bib-0028] Cortese, S. , Solmi, M. , Michelini, G. , Bellato, A. , Blanner, C. , Canozzi, A. , Eudave, L. , Farhat, L. C. , Højlund, M. , Köhler‐Forsberg, O. , Leffa, D. T. , Rohde, C. , de Pablo, G. S. , Vita, G. , Wesselhoeft, R. , Martin, J. , Baumeister, S. , Bozhilova, N. S. , Carlisi, C. O. , & Correll, C. U. (2023). Candidate diagnostic biomarkers for neurodevelopmental disorders in children and adolescents: A systematic review. World Psychiatry, 22(1), 129–149. 10.1002/wps.21037 36640395 PMC9840506

[jcv270142-bib-0029] Costello, E. J. , Mustillo, S. , Erkanli, A. , Keeler, G. , & Angold, A. (2003). Prevalence and development of psychiatric disorders in childhood and adolescence. Archives of General Psychiatry, 60(8), 837–844. 10.1001/archpsyc.60.8.837 12912767

[jcv270142-bib-0030] Cramer, A. O. J. , Waldorp, L. J. , Maas, H. L. J. van der , & Borsboom, D. (2010). Comorbidity: A network perspective. Behavioral and Brain Sciences, 33(2–3), 137–150. 10.1017/S0140525X09991567 20584369

[jcv270142-bib-0031] Curran, P. J. , Howard, A. L. , Bainter, S. A. , Lane, S. T. , & McGinley, J. S. (2014). The separation of between‐person and within‐person components of individual change over time: A latent curve model with structured residuals. Journal of Consulting and Clinical Psychology, 82(5), 879–894. 10.1037/a0035297 24364798 PMC4067471

[jcv270142-bib-0032] Dalsgaard, S. , Thorsteinsson, E. , Trabjerg, B. B. , Schullehner, J. , Plana‐Ripoll, O. , Brikell, I. , Wimberley, T. , Thygesen, M. , Madsen, K. B. , Timmerman, A. , Schendel, D. , McGrath, J. J. , Mortensen, P. B. , & Pedersen, C. B. (2020). Incidence rates and cumulative incidences of the full spectrum of diagnosed mental disorders in childhood and adolescence. JAMA Psychiatry, 77(2), 155–164. 10.1001/jamapsychiatry.2019.3523 31746968 PMC6902162

[jcv270142-bib-0033] De Los Reyes, A. , Augenstein, T. M. , Wang, M. , Thomas, S. A. , Drabick, D. A. G. , Burgers, D. E. , & Rabinowitz, J. (2015). The validity of the multi‐informant approach to assessing child and adolescent mental health. Psychological Bulletin, 141(4), 858–900. 10.1037/a0038498 25915035 PMC4486608

[jcv270142-bib-0034] Dormann, C. , & Griffin, M. A. (2015). Optimal time lags in panel studies. Psychological Methods, 20(4), 489–505. 10.1037/met0000041 26322999

[jcv270142-bib-0035] Du Rietz, E. , Pettersson, E. , Brikell, I. , Ghirardi, L. , Chen, Q. , Hartman, C. , Lichtenstein, P. , Larsson, H. , & Kuja‐Halkola, R. (2021). Overlap between attention‐deficit hyperactivity disorder and neurodevelopmental, externalising and internalising disorders: Separating unique from general psychopathology effects. The British Journal of Psychiatry, 218(1), 35–42. 10.1192/bjp.2020.152 32892757

[jcv270142-bib-0036] Edwards, A. C. , & Kendler, K. S. (2012). Twin study of the relationship between adolescent attention‐deficit/hyperactivity disorder and adult alcohol dependence. Journal of Studies on Alcohol and Drugs, 73(2), 185–194. 10.15288/jsad.2012.73.185 22333326 PMC3281978

[jcv270142-bib-0037] Eley, T. C. (1997). General genes: A new theme in developmental psychopathology. Current Directions in Psychological Science, 6(4), 90–95. 10.1111/1467-8721.ep11512831

[jcv270142-bib-0038] Elkins, I. J. , King, S. M. , McGue, M. , & Iacono, W. G. (2006). Personality traits and the development of nicotine, alcohol, and illicit drug disorders: Prospective links from adolescence to young adulthood. Journal of Abnormal Psychology, 115(1), 26–39. 10.1037/0021-843X.115.1.26 16492093

[jcv270142-bib-0039] Evans, S. C. , Fite, P. J. , Hendrickson, M. L. , Rubens, S. L. , & Mages, A. K. (2015). The role of reactive aggression in the link between hyperactive–impulsive behaviors and peer rejection in adolescents. Child Psychiatry and Human Development, 46(6), 903–912. 10.1007/s10578-014-0530-y 25552242

[jcv270142-bib-0040] Farhat, L. C. , Brentani, H. , de Toledo, V. H. C. , Shephard, E. , Mattos, P. , Baron‐Cohen, S. , Thapar, A. , Casella, E. , & Polanczyk, G. V. (2022). ADHD and autism symptoms in youth: A network analysis. Journal of Child Psychology and Psychiatry, 63(2), 143–151. 10.1111/jcpp.13436 33984874

[jcv270142-bib-0041] Feeley, T. H. (2002). Comment on halo effects in rating and evaluation research. Human Communication Research, 28(4), 578–586. 10.1111/j.1468-2958.2002.tb00825.x

[jcv270142-bib-0042] Forgas, J. P. , & Laham, S. M. (2016). Halo effects. In Cognitive illusions (2nd ed.). Psychology Press.

[jcv270142-bib-0043] Fried, E. I. (2020). Lack of theory building and testing impedes progress in the factor and network literature. Psychological Inquiry, 31(4), 271–288. 10.1080/1047840X.2020.1853461

[jcv270142-bib-0044] Fried, E. I. , & Cramer, A. O. J. (2017). Moving forward: Challenges and directions for psychopathological network theory and methodology. Perspectives on Psychological Science, 12(6), 999–1020. 10.1177/1745691617705892 28873325

[jcv270142-bib-0045] Funder, D. C. , & Ozer, D. J. (2019). Evaluating effect size in psychological research: Sense and nonsense. Advances in Methods and Practices in Psychological Science, 2(2), 156–168. 10.1177/2515245919847202

[jcv270142-bib-0046] Glessner, J. T. , Li, J. , Wang, D. , March, M. , Lima, L. , Desai, A. , Hadley, D. , Kao, C. , Gur, R. E. , Cohen, N. , Sleiman, P. M. A. , Li, Q. , Hakonarson, H. , Sleiman, P. , Glessner, J. , Hadley, D. , Kao, C. , Wu, Z. , Kim, C. , & Li, Q. (2017). Copy number variation meta‐analysis reveals a novel duplication at 9p24 associated with multiple neurodevelopmental disorders. Genome Medicine, 9(1), 106. 10.1186/s13073-017-0494-1 29191242 PMC5709845

[jcv270142-bib-0047] Goulter, N. , McMahon, R. J. , Lansford, J. E. , Bates, J. E. , Dodge, K. A. , Crowley, D. M. , & Pettit, G. S. (2022). Externalizing psychopathology from childhood to early adolescence: Psychometric evaluation using latent variable and network modeling. Psychological Assessment, 34(11), 1008–1021. 10.1037/pas0001163 36074612 PMC10040489

[jcv270142-bib-0048] Griep, Y. , Vranjes, I. , Kraak, J. M. , Dudda, L. , & Li, Y. (2021). Start small, not random: Why does justifying your time‐lag matter? Spanish Journal of Psychology, 24, e45. 10.1017/SJP.2021.42 34511144

[jcv270142-bib-0049] Hawkins, E. , Gathercole, S. , Astle, D. , Calm Team , & Holmes, J. (2016). Language problems and ADHD symptoms: How specific are the links? Brain Sciences, 6(4), 50. 10.3390/brainsci6040050 27775648 PMC5187564

[jcv270142-bib-0050] Hettwer, M. D. , Larivière, S. , Park, B. Y. , van den Heuvel, O. A. , Schmaal, L. , Andreassen, O. A. , Ching, C. R. K. , Hoogman, M. , Buitelaar, J. , van Rooij, D. , Veltman, D. J. , Stein, D. J. , Franke, B. , van Erp, T. G. M. , Jahanshad, N. , Thompson, P. M. , Thomopoulos, S. I. , Bethlehem, R. A. I. , Bernhardt, B. C. , & Valk, S. L. (2022). Coordinated cortical thickness alterations across six neurodevelopmental and psychiatric disorders. Nature Communications, 13(1), 6851. 10.1038/s41467-022-34367-6 PMC9652311 36369423

[jcv270142-bib-0051] Hicks, B. M. , Krueger, R. F. , Iacono, W. G. , McGue, M. , & Patrick, C. J. (2004). Family transmission and heritability of externalizing disorders: A twin‐family study. Archives of General Psychiatry, 61(9), 922–928. 10.1001/archpsyc.61.9.922 15351771

[jcv270142-bib-0052] Hinshaw, S. P. , Owens, E. B. , Zalecki, C. , Huggins, S. P. , Montenegro‐Nevado, A. J. , Schrodek, E. , & Swanson, E. N. (2012). Prospective follow‐up of girls with attention‐deficit/hyperactivity disorder into early adulthood: Continuing impairment includes elevated risk for suicide attempts and self‐injury. Journal of Consulting and Clinical Psychology, 80(6), 1041–1051. 10.1037/a0029451 22889337 PMC3543865

[jcv270142-bib-0053] Hoffman, L. , & Stawski, R. S. (2009). Persons as contexts: Evaluating between‐person and within‐person effects in longitudinal analysis. Research in Human Development, 6(2–3), 97–120. 10.1080/15427600902911189

[jcv270142-bib-0054] Hoffmann, W. , Weber, L. , König, U. , Becker, K. , & Kamp‐Becker, I. (2016). The role of the CBCL in the assessment of autism spectrum disorders: An evaluation of symptom profiles and screening characteristics. Research in Autism Spectrum Disorders, 27, 44–53. 10.1016/j.rasd.2016.04.002

[jcv270142-bib-0055] Holmes, J. , Mareva, S. , Bennett, M. P. , Black, M. J. , & Guy, J. (2021). Higher‐order dimensions of psychopathology in a neurodevelopmental transdiagnostic sample. Journal of Abnormal Psychology, 130(8), 909–922. 10.1037/abn0000710 34843293 PMC8628482

[jcv270142-bib-0056] Karalunas, S. L. , Antovich, D. , Goh, P. K. , Martel, M. M. , Tipsord, J. , Nousen, E. K. , & Nigg, J. T. (2021). Longitudinal network model of the co‐development of temperament, executive functioning, and psychopathology symptoms in youth with and without ADHD. Development and Psychopathology, 33(5), 1803–1820. 10.1017/S0954579421000900 35210712 PMC8863133

[jcv270142-bib-0057] Kendler, K. S. , Prescott, C. A. , Myers, J. , & Neale, M. C. (2003). The structure of genetic and environmental risk factors for common psychiatric and substance use disorders in men and women. Archives of General Psychiatry, 60(9), 929–937. 10.1001/archpsyc.60.9.929 12963675

[jcv270142-bib-0058] Kirisci, L. , Tarter, R. E. , Reynolds, M. , & Vanyukov, M. (2006). Individual differences in childhood neurobehavior disinhibition predict decision to desist substance use during adolescence and substance use disorder in young adulthood: A prospective study. Addictive Behaviors, 31(4), 686–696. 10.1016/j.addbeh.2005.05.049 15964148

[jcv270142-bib-0059] Koo, T. K. , & Li, M. Y. (2016). A guideline of selecting and reporting intraclass correlation coefficients for reliability research. Journal of Chiropractic Medicine, 15(2), 155–163. 10.1016/j.jcm.2016.02.012 27330520 PMC4913118

[jcv270142-bib-0060] Kotov, R. , Krueger, R. F. , Watson, D. , Cicero, D. C. , Conway, C. C. , DeYoung, C. G. , Eaton, N. R. , Forbes, M. K. , Hallquist, M. N. , Latzman, R. D. , Mullins‐Sweatt, S. N. , Ruggero, C. J. , Simms, L. J. , Waldman, I. D. , Waszczuk, M. A. , & Wright, A. G. C. (2021). The hierarchical taxonomy of psychopathology (HiTOP): A quantitative nosology based on consensus of evidence. Annual Review of Clinical Psychology, 17(1), 83–108. 10.1146/annurev-clinpsy-081219-093304 33577350

[jcv270142-bib-0061] Kovas, Y. , & Plomin, R. (2007). Learning abilities and disabilities: Generalist genes, specialist environments. Current Directions in Psychological Science, 16(5), 284–288. 10.1111/j.1467-8721.2007.00521.x 20351764 PMC2841819

[jcv270142-bib-0062] Krueger, R. F. (1999a). The structure of common mental disorders. Archives of General Psychiatry, 56(10), 921–926. 10.1001/archpsyc.56.10.921 10530634

[jcv270142-bib-0063] Krueger, R. F. (1999b). The structure of common mental disorders. Archives of General Psychiatry, 56(10), 921–926. 10.1001/archpsyc.56.10.921 10530634

[jcv270142-bib-0064] Krueger, R. F. , Hicks, B. M. , Patrick, C. J. , Carlson, S. R. , Iacono, W. G. , & McGue, M. (2002). Etiologic connections among substance dependence, antisocial behavior, and personality: Modeling the externalizing spectrum. Journal of Abnormal Psychology, 111(3), 411–424. 10.1037/0021-843x.111.3.411 12150417

[jcv270142-bib-0065] Krueger, R. F. , Hobbs, K. A. , Conway, C. C. , Dick, D. M. , Dretsch, M. N. , Eaton, N. R. , Forbes, M. K. , Forbush, K. T. , Keyes, K. M. , Latzman, R. D. , Michelini, G. , Patrick, C. J. , Sellbom, M. , Slade, T. , South, S. C. , Sunderland, M. , Tackett, J. , Waldman, I. , Waszczuk, M. A. , & Kotov, R. (2021). Validity and utility of hierarchical taxonomy of psychopathology (HiTOP): II. Externalizing superspectrum. World Psychiatry, 20(2), 171–193. 10.1002/wps.20844 34002506 PMC8129870

[jcv270142-bib-0066] Krueger, R. F. , Kotov, R. , Watson, D. , Forbes, M. K. , Eaton, N. R. , Ruggero, C. J. , Simms, L. J. , Widiger, T. A. , Achenbach, T. M. , Bach, B. , Bagby, R. M. , Bornovalova, M. A. , Carpenter, W. T. , Chmielewski, M. , Cicero, D. C. , Clark, L. A. , Conway, C. , DeClercq, B. , DeYoung, C. G. , & Zimmermann, J. (2018). Progress in achieving quantitative classification of psychopathology. World Psychiatry, 17(3), 282–293. 10.1002/wps.20566 30229571 PMC6172695

[jcv270142-bib-0067] Krueger, R. F. , & Markon, K. E. (2006). Reinterpreting comorbidity: A model‐based approach to understanding and classifying psychopathology. Annual Review of Clinical Psychology, 2(1), 111–133. 10.1146/annurev.clinpsy.2.022305.095213 PMC2242354 17716066

[jcv270142-bib-0068] Krueger, R. F. , Markon, K. E. , Patrick, C. J. , & Iacono, W. G. (2005). Externalizing psychopathology in adulthood: A dimensional‐spectrum conceptualization and its implications for DSM–V. Journal of Abnormal Psychology, 114(4), 537–550. 10.1037/0021-843X.114.4.537 16351376 PMC2242352

[jcv270142-bib-0069] Laceulle, O. M. , Vollebergh, W. A. , & Ormel, J. (2015). The structure of psychopathology in adolescence: Replication of a general psychopathology factor in the TRAILS study. Clinical Psychological Science, 3(6), 850–860. 10.1177/2167702614560750

[jcv270142-bib-0070] Lee, S. S. , Humphreys, K. L. , Flory, K. , Liu, R. , & Glass, K. (2011). Prospective association of childhood attention‐deficit/hyperactivity disorder (ADHD) and substance use and abuse/dependence: A meta‐analytic review. Clinical Psychology Review, 31(3), 328–341. 10.1016/j.cpr.2011.01.006 21382538 PMC3180912

[jcv270142-bib-0071] Leopold, D. R. , Christopher, M. E. , Burns, G. L. , Becker, S. P. , Olson, R. K. , & Willcutt, E. G. (2016). Attention‐deficit/hyperactivity disorder and sluggish cognitive tempo throughout childhood: Temporal invariance and stability from preschool through ninth grade. Journal of Child Psychology and Psychiatry, 57(9), 1066–1074. 10.1111/jcpp.12505 26749438 PMC4938772

[jcv270142-bib-0126] Leopold, D. R. , Christopher, M. E. , Olson, R. K. , Petrill, S. A. , & Willcutt, E. G. (2019). Invariance of ADHD symptoms across sex and age: A latent analysis of ADHD and impairment ratings from early childhood into adolescence. Journal of Abnormal Child Psychology, 47(1), 21–34. 29691720 10.1007/s10802-018-0434-6 PMC6202270

[jcv270142-bib-0072] Lilienfeld, S. O. , Waldman, I. D. , & Israel, A. C. (1994). A critical examination of the use of the term and concept of comorbidity in psychopathology research. Clinical Psychology: Science and Practice, 1(1), 71–83. 10.1111/j.1468-2850.1994.tb00007.x

[jcv270142-bib-0073] Liu, K. , Thompson, R. C. , Watson, J. , Montena, A. L. , & Warren, S. L. (2023). Developmental trajectories of internalizing and externalizing symptoms in youth and associated gender differences: A directed network perspective. Research on Child and Adolescent Psychopathology, 51(11), 1627–1639. 10.1007/s10802-023-01106-4 37548898 PMC10627904

[jcv270142-bib-0074] Lonigan, C. J. , Phillips, B. M. , & Hooe, E. S. (2003). Relations of positive and negative affectivity to anxiety and depression in children: Evidence from a latent variable longitudinal study. Journal of Consulting and Clinical Psychology, 71(3), 465–481. 10.1037/0022-006x.71.3.465 12795571

[jcv270142-bib-0075] Martel, M. M. , Levinson, C. A. , Lee, C. A. , & Smith, T. E. (2017). Impulsivity symptoms as core to the developmental externalizing spectrum. Journal of Abnormal Child Psychology, 45(1), 83–90. 10.1007/s10802-016-0148-6 27017822 PMC5040618

[jcv270142-bib-0076] Martel, M. M. , Roberts, B. , Gremillion, M. , von Eye, A. , & Nigg, J. T. (2011). External validation of bifactor model of ADHD: Explaining heterogeneity in psychiatric comorbidity, cognitive control, and personality trait profiles within DSM‐IV ADHD. Journal of Abnormal Child Psychology, 39(8), 1111–1123. 10.1007/s10802-011-9538-y 21735050 PMC3199328

[jcv270142-bib-0077] McCutcheon, R. A. , Pillinger, T. , Guo, X. , Rogdaki, M. , Welby, G. , Vano, L. , Cummings, C. , Heron, T.‐A. , Brugger, S. , Davies, D. , Ghanem, M. , Efthimiou, O. , Cipriani, A. , & Howes, O. D. (2023). Shared and separate patterns in brain morphometry across transdiagnostic dimensions. Nature Mental Health, 1(1), 55–65. 10.1038/s44220-022-00010-y

[jcv270142-bib-0078] McElroy, E. , Belsky, J. , Carragher, N. , Fearon, P. , & Patalay, P. (2018). Developmental stability of general and specific factors of psychopathology from early childhood to adolescence: Dynamic mutualism or p‐differentiation? Journal of Child Psychology and Psychiatry, 59(6), 667–675. 10.1111/jcpp.12849 29197107 PMC6001631

[jcv270142-bib-0079] McElroy, E. , Shevlin, M. , Murphy, J. , & McBride, O. (2018). Co‐occurring internalizing and externalizing psychopathology in childhood and adolescence: A network approach. European Child & Adolescent Psychiatry, 27(11), 1449–1457. 10.1007/s00787-018-1128-x 29520540

[jcv270142-bib-0080] McLaughlin, K. A. , & Hatzenbuehler, M. L. (2009). Stressful life events, anxiety sensitivity, and internalizing symptoms in adolescents. Journal of Abnormal Psychology, 118(3), 659–669. 10.1037/a0016499 19685962 PMC2881589

[jcv270142-bib-0081] McNally, R. J. (2021). Network analysis of psychopathology: Controversies and challenges. Annual Review of Clinical Psychology, 17(1), 31–53. 10.1146/annurev-clinpsy-081219-092850 33228401

[jcv270142-bib-0082] Michelini, G. , Barch, D. M. , Tian, Y. , Watson, D. , Klein, D. N. , & Kotov, R. (2019). Delineating and validating higher‐order dimensions of psychopathology in the adolescent brain cognitive development (ABCD) study. Translational Psychiatry, 9(1), 261. Article 1. 10.1038/s41398-019-0593-4 31624235 PMC6797772

[jcv270142-bib-0083] Michelini, G. , Carlisi, C. O. , Eaton, N. R. , Elison, J. T. , Haltigan, J. D. , Kotov, R. , Krueger, R. F. , Latzman, R. D. , Li, J. J. , Levin‐Aspenson, H. F. , Salum, G. A. , South, S. C. , Stanton, K. , Waldman, I. D. , & Wilson, S. (2024). Where do neurodevelopmental conditions fit in transdiagnostic psychiatric frameworks? Incorporating a new neurodevelopmental spectrum. World Psychiatry, 23(3), 333–357. 10.1002/wps.21225 39279404 PMC11403200

[jcv270142-bib-0084] Mrug, S. , Molina, B. S. G. , Hoza, B. , Gerdes, A. C. , Hinshaw, S. P. , Hechtman, L. , & Arnold, L. E. (2012). Peer rejection and friendships in children with attention‐deficit/hyperactivity disorder: Contributions to long‐term outcomes. Journal of Abnormal Child Psychology, 40(6), 1013–1026. 10.1007/s10802-012-9610-2 22331455 PMC3384771

[jcv270142-bib-0085] Murray, A. L. , Eisner, M. , & Ribeaud, D. (2016). The development of the general factor of psychopathology ‘p factor’through childhood and adolescence. Journal of Abnormal Child Psychology, 44(8), 1573–1586. 10.1007/s10802-016-0132-1 26846993

[jcv270142-bib-0086] Muthén, L. K. , & Muthén, B. (2020). Mplus (version 8.5) [Computer software]. Muthén & Muthén. Retrieved from https://www.statmodel.com/index.shtml

[jcv270142-bib-0087] Nadder, T. S. , Rutter, M. , Silberg, J. L. , Maes, H. H. , & Eaves, L. J. (2002). Genetic effects on the variation and covariation of attention deficit‐hyperactivity disorder (ADHD) and oppositional‐defiant disorder/conduct disorder (ODD/CD) symptomatologies across informant and occasion of measurement. Psychological Medicine, 32(1), 39–53. 10.1017/S0033291701004792 11883729

[jcv270142-bib-0088] Neuhaus, E. , & Beauchaine, T. P. (2017). Impulsivity and vulnerability to psychopathology. Child and Adolescent Psychopathology. 178–212.

[jcv270142-bib-0089] Nigg, J. T. , Sibley, M. H. , Thapar, A. , & Karalunas, S. L. (2020). Development of ADHD: Etiology, heterogeneity, and early life course. Annual Review of Developmental Psychology, 2(1), 559–583. 10.1146/annurev-devpsych-060320-093413 PMC8336725 34368774

[jcv270142-bib-0090] Noordhof, A. , Krueger, R. F. , Ormel, J. , Oldehinkel, A. J. , & Hartman, C. A. (2015). Integrating autism‐related symptoms into the dimensional internalizing and externalizing model of psychopathology. The TRAILS study. Journal of Abnormal Child Psychology, 43(3), 577–587. 10.1007/s10802-014-9923-4 25099360

[jcv270142-bib-0091] Obsuth, I. , Murray, A. L. , Di Folco, S. , Ribeaud, D. , & Eisner, M. (2020). Patterns of homotypic and heterotypic continuity between ADHD symptoms, externalising and internalising problems from age 7 to 15. Journal of Abnormal Child Psychology, 48(2), 223–236. 10.1007/s10802-019-00592-9 31705348 PMC6969859

[jcv270142-bib-0092] Ooi, Y. P. , Rescorla, L. , Ang, R. P. , Woo, B. , & Fung, D. S. S. (2011). Identification of autism spectrum disorders using the child behavior checklist in Singapore. Journal of Autism and Developmental Disorders, 41(9), 1147–1156. 10.1007/s10803-010-1015-x 20405192

[jcv270142-bib-0093] Orth, U. , Clark, D. A. , Donnellan, M. B. , & Robins, R. W. (2021). Testing prospective effects in longitudinal research: Comparing seven competing cross‐lagged models. Journal of Personality and Social Psychology, 120(4), 1013–1034. 10.1037/pspp0000358 32730068 PMC7854859

[jcv270142-bib-0094] Patterson, G. R. (1995). Orderly change in a stable world: The antisocial trait as a chimera. In J. M. Gottman (Ed.), The analysis of change (pp. 141–182). Psychology Press. 10.1037//0022-006x.61.6.911 8113492

[jcv270142-bib-0095] Peng, Z. , Nguyen, P. T. , Hinshaw, S. , & Watts, A. L. (n.d.). A comprehensive examination of sex‐differentiated correlates of ADHD symptoms in late childhood and early adolescence. Clinical Psychological Science. https://osf.io/n7uka 10.1177/21677026261454238 PMC13557031 42719335

[jcv270142-bib-0096] Peng, Z. , Stanton, K. , Dominguez‐Alvarez, B. , & Watts, A. L. (2025). Where does attention‐deficit/hyperactivity disorder fit in the psychopathology hierarchy? A symptom‐focused analysis. Journal of Psychopathology and Clinical Science, 134(2), 143–161. 10.1037/abn0000966 PMC12226816 39679963

[jcv270142-bib-0097] Perkins, E. R. , Joyner, K. J. , Foell, J. , Drislane, L. E. , Brislin, S. J. , Frick, P. J. , Yancey, J. R. , Soto, E. F. , Ganley, C. M. , Keel, P. K. , Sica, C. , Flor, H. , Nees, F. , Banaschewski, T. , Bokde, A. L. W. , Desrivières, S. , Grigis, A. , Garavan, H. , Gowland, P. , & Imagen Consortium . (2022). Assessing general versus specific liability for externalizing problems in adolescence: Concurrent and prospective prediction of symptoms of conduct disorder, ADHD, and substance use. Journal of Psychopathology and Clinical Science, 131(7), 793–807. 10.1037/abn0000743 36222627 PMC9710196

[jcv270142-bib-0098] Pettersson, E. , Anckarsäter, H. , Gillberg, C. , & Lichtenstein, P. (2013). Different neurodevelopmental symptoms have a common genetic etiology. Journal of Child Psychology and Psychiatry, 54(12), 1356–1365. 10.1111/jcpp.12113 24127638

[jcv270142-bib-0099] Pettersson, E. , Larsson, H. , D’Onofrio, B. M. , & Lichtenstein, P. (2023). Associations between general and specific psychopathology factors and 10‐year clinically relevant outcomes in adult Swedish twins and siblings. JAMA Psychiatry, 80(7), 728–737. 10.1001/jamapsychiatry.2023.1162 37163290 PMC10173102

[jcv270142-bib-0100] Ralf, K.‐H. , Paul, L. , Brian, M. D. , & Henrik, L. (2015). Co‐development of ADHD and externalizing behavior from childhood to adulthood. The Journal of Child Psychology and Psychiatry and Allied Disciplines, 56(6), 640–647. 10.1111/jcpp.12340 25303006 PMC4393334

[jcv270142-bib-0101] R Core Team . (2023). R: A language and environment for statistical computing [Computer software]. R Foundation for Statistical Computing. Retrieved from https://www.R‐project.org/

[jcv270142-bib-0102] Ribasés, M. , Mitjans, M. , Hartman, C. , Artigas, M. S. , Demontis, D. , Larsson, H. , Ramos‐Quiroga, J. , Kuntsi, J. , Faraone, S. , Børglum, A. , Reif, A. , Franke, B. , & Cormand, B. (2023). Genetic architecture of ADHD and overlap with other psychiatric disorders and cognition‐related phenotypes. Neuroscience & Biobehavioral Reviews, 153, 105313. 10.1016/j.neubiorev.2023.105313 37451654 PMC10789879

[jcv270142-bib-0103] Richards, J. S. , Hartman, C. A. , Ormel, J. , & Oldehinkel, A. J. (2024). Continuity of psychopathology throughout adolescence and young adulthood. Journal of Clinical Child and Adolescent Psychology, 53(4), 623–636. 10.1080/15374416.2022.2042695 35259007 PMC11318507

[jcv270142-bib-0104] Robinaugh, D. J. , Haslbeck, J. M. B. , Ryan, O. , Fried, E. I. , & Waldorp, L. J. (2021). Invisible hands and fine calipers: A call to use formal theory as a toolkit for theory construction. Perspectives on Psychological Science, 16(4), 725–743. 10.1177/1745691620974697 33593176 PMC8273080

[jcv270142-bib-0105] Rondon, A. T. , Hilton, D. C. , Jarrett, M. A. , & Ollendick, T. H. (2020). Sleep, internalizing problems, and social withdrawal: Unique associations in clinic‐referred youth with elevated sluggish cognitive tempo symptoms. Journal of Attention Disorders, 24(4), 524–534. 10.1177/1087054718756197 29415601

[jcv270142-bib-0106] Servera, M. , Sáez, B. , Burns, G. L. , & Becker, S. P. (2018). Clinical differentiation of sluggish cognitive tempo and attention‐deficit/hyperactivity disorder in children. Journal of Abnormal Psychology, 127(8), 818–829. 10.1037/abn0000375 30265014 PMC6237634

[jcv270142-bib-0107] Silk, T. J. , Malpas, C. B. , Beare, R. , Efron, D. , Anderson, V. , Hazell, P. , Jongeling, B. , Nicholson, J. M. , & Sciberras, E. (2019). A network analysis approach to ADHD symptoms: More than the sum of its parts. PLoS One, 14(1), e0211053. 10.1371/journal.pone.0211053 30657783 PMC6338383

[jcv270142-bib-0108] So, P. , Greaves‐Lord, K. , van der Ende, J. , Verhulst, F. C. , Rescorla, L. , & de Nijs, P. F. (2013). Using the child behavior checklist and the Teacher’s report form for identification of children with autism spectrum disorders. Autism, 17(5), 595–607. 10.1177/1362361312448855 22914776

[jcv270142-bib-0109] Spearman, C. (1904). “General intelligence,” objectively determined and measured. American Journal of Psychology, 15(2), 201–293. 10.2307/1412107

[jcv270142-bib-0110] Spearman, C. (1961). “General intelligence” objectively determined and measured (p. 73). Appleton‐Century‐Crofts. 10.1037/11491-006

[jcv270142-bib-0111] Speyer, L. G. , Eisner, M. , Ribeaud, D. , Luciano, M. , Auyeung, B. , & Murray, A. L. (2021). Developmental relations between internalising problems and ADHD in childhood: A symptom level perspective. Research on Child and Adolescent Psychopathology, 49(12), 1567–1579. 10.1007/s10802-021-00856-3 34363556 PMC8557182

[jcv270142-bib-0112] Sterba, S. K. , Copeland, W. , Egger, H. L. , Jane Costello, E. , Erkanli, A. , & Angold, A. (2010). Longitudinal dimensionality of adolescent psychopathology: Testing the differentiation hypothesis. Journal of Child Psychology and Psychiatry, 51(8), 871–884. 10.1111/j.1469-7610.2010.02234.x 20345843 PMC3630513

[jcv270142-bib-0113] Sturm, A. , Kuhfeld, M. , Kasari, C. , & McCracken, J. T. (2017). Development and validation of an item response theory‐based social responsiveness scale short form. Journal of Child Psychology and Psychiatry, 58(9), 1053–1061. 10.1111/jcpp.12731 28464350

[jcv270142-bib-0114] Tarter, R. E. , Kirisci, L. , Habeych, M. , Reynolds, M. , & Vanyukov, M. (2004). Neurobehavior disinhibition in childhood predisposes boys to substance use disorder by young adulthood: Direct and mediated etiologic pathways. Drug and Alcohol Dependence, 73(2), 121–132. 10.1016/j.drugalcdep.2003.07.004 14725951

[jcv270142-bib-0115] Thapar, A. , Cooper, M. , & Rutter, M. (2017). Neurodevelopmental disorders. The Lancet Psychiatry, 4(4), 339–346. 10.1016/S2215-0366(16)30376-5 27979720

[jcv270142-bib-0116] Uhlhaas, P. J. , Davey, C. G. , Mehta, U. M. , Shah, J. , Torous, J. , Allen, N. B. , Avenevoli, S. , Bella‐Awusah, T. , Chanen, A. , Chen, E. Y. H. , Correll, C. U. , Do, K. Q. , Fisher, H. L. , Frangou, S. , Hickie, I. B. , Keshavan, M. S. , Konrad, K. , Lee, F. S. , Liu, C. H. , & Wood, S. J. (2023). Towards a youth mental health paradigm: A perspective and roadmap. Molecular Psychiatry, 28(8), 3171–3181. 10.1038/s41380-023-02202-z 37580524 PMC10618105

[jcv270142-bib-0117] Van Bork, R. , Epskamp, S. , Rhemtulla, M. , Borsboom, D. , & van der Maas, H. L. (2017). What is the p‐factor of psychopathology? Some risks of general factor modeling. Theory & Psychology, 27(6), 759–773. 10.1177/0959354317737185

[jcv270142-bib-0118] Van Der Maas, H. L. J. , Dolan, C. V. , Grasman, R. P. P. P. , Wicherts, J. M. , Huizenga, H. M. , & Raijmakers, M. E. J. (2006). A dynamical model of general intelligence: The positive manifold of intelligence by mutualism. Psychological Review, 113(4), 842–861. 10.1037/0033-295X.113.4.842 17014305

[jcv270142-bib-0119] Van der Stel, M. , & Veenman, M. V. J. (2010). Development of metacognitive skillfulness: A longitudinal study. Learning and Individual Differences, 20(3), 220–224. 10.1016/j.lindif.2009.11.005

[jcv270142-bib-0120] Van Meter, A. R. , Sibley, M. H. , Vandana, P. , Birmaher, B. , Fristad, M. A. , Horwitz, S. , Youngstrom, E. A. , Findling, R. L. , & Arnold, L. E. (2024). The stability and persistence of symptoms in childhood‐onset ADHD. European Child & Adolescent Psychiatry, 33(4), 1163–1170. 10.1007/s00787-023-02235-3 37270740 PMC12490748

[jcv270142-bib-0121] Vergunst, F. , Tremblay, R. E. , Galera, C. , Nagin, D. , Vitaro, F. , Boivin, M. , & Côté, S. M. (2019). Multi‐rater developmental trajectories of hyperactivity–impulsivity and inattention symptoms from 1.5 to 17 years: A population‐based birth cohort study. European Child & Adolescent Psychiatry, 28(7), 973–983. 10.1007/s00787-018-1258-1 30506420 PMC6647515

[jcv270142-bib-0122] Waszczuk, M. A. , Miao, J. , Docherty, A. R. , Shabalin, A. A. , Jonas, K. G. , Michelini, G. , & Kotov, R. (2023). General v. Specific vulnerabilities: Polygenic risk scores and higher‐order psychopathology dimensions in the adolescent brain cognitive development (ABCD) study. Psychological Medicine, 53(5), 1937–1946. 10.1017/S0033291721003639 37310323 PMC10958676

[jcv270142-bib-0123] Weil, L. G. , Fleming, S. M. , Dumontheil, I. , Kilford, E. J. , Weil, R. S. , Rees, G. , Dolan, R. J. , & Blakemore, S.‐J. (2013). The development of metacognitive ability in adolescence. Consciousness and Cognition, 22(1), 264–271. 10.1016/j.concog.2013.01.004 23376348 PMC3719211

[jcv270142-bib-0124] World Health Organization . (2022). In International classification of diseases eleventh revision (ICD‐11). World Health Organization. License: CC BY‐ND 3.0 IGO.

[jcv270142-bib-0125] Young, S. E. , Stallings, M. C. , Corley, R. P. , Krauter, K. S. , & Hewitt, J. K. (2000). Genetic and environmental influences on behavioral disinhibition. American Journal of Medical Genetics, 96(5), 684–695. 10.1002/1096-8628(20001009)96:5<684::AID-AJMG16>3.0.CO;2-G 11054778

