# Supplementary material for: Common cause versus dynamic mutualism: Insights into ADHD's common comorbidities
Source: JCPP Adv. 2026 Jun 18:e70142. Online ahead of print. doi: 10.1002/jcv2.70142 (PMC13339281; doi:10.1002/jcv2.70142)
Supplement: Supplementary file 1 — Supporting Information S1 [file JCV2-9999-e70142-s002.docx]

**Common Cause versus Dynamic Mutualism:**

**Insights into ADHD’s Common Comorbidities**

**Supporting Information**

**Appendix S1. Evidence for Sex differences**

We examined sex differences using multi-group modeling, in which boys and girls were specified as separate groups. Differences between the two sexes were inferred by comparing group-specific parameter estimates and their 83% confidence intervals (CIs). We used an 83% CI rather than a 95% CI to avoid overly stringent comparisons (Payton et al., 2003; Schenker & Gentleman, 2001).^[[1]](#footnote-1)^

Overall, evidence for sex differences was limited. First, the random intercepts for ADHD and its subdimensions (i.e., inattention, hyperactivity/impulsivity) were more strongly associated with both externalizing and neurodevelopmental spectra in boys than in girls. Still, this difference in magnitude did not alter the overall pattern of results, which continued to provide strong support for a common-cause account in both sexes. Second, the random intercepts and slopes for ADHD were more strongly associated with those for the neurodevelopmental spectrum than the externalizing spectrum in boys, whereas this pattern was not observed in girls. In fact, the random intercept for ADHD was more strongly associated with the externalizing spectrum than the neurodevelopmental spectrum in girls (Table S4). Although this finding may be interesting to explore in future research, it deviates from the primary aim of the current study, which was to adjudicate between common-cause and dynamic mutualism models in explaining the developmental links between ADHD and the neurodevelopmental versus externalizing spectra, to inform ADHD classification.

As such, we present detailed evidence for both the common-cause and dynamic mutualism models below rather than in the main text. A summary of the results is provided in Table S4.

**Evidence for the Common Cause Model Across Sexes**

***Externalizing Spectrum***

**Boys.** The random intercepts for ADHD and the externalizing spectrum were strongly correlated (*r* = .82), whereas the random slopes were moderately negatively correlated (*r* = -.44). These correlations varied across ADHD subdimensions.

Correlations between random intercepts for ADHD subdimensions and the externalizing spectrum were very large for inattention (*r* = .77) and cognitive disengagement (*r* = .58). Random slopes for ADHD subdimensions were very strongly negatively correlated with those for the externalizing spectrum (*r*s = -.81 for both inattention and cognitive disengagement).

**Girls.** The random intercepts for ADHD and the externalizing spectrum were very strongly correlated (*r* = .66), as were the random slopes (*r* = .70). Correlation patterns varied across ADHD subdimensions (i.e., inattention and cognitive disengagement).

Correlations between random intercepts of ADHD subdimensions and the externalizing spectrum were very large for inattention (*r* = .55) and cognitive disengagement (*r* = .40). Likewise, correlations between random slopes of ADHD subdimensions and the externalizing spectrum were very large for inattention (*r* = .61) and cognitive disengagement (*r* = .47).

***Neurodevelopmental Spectrum***

**Boys.** The random intercepts for ADHD and the neurodevelopmental spectrum were very strongly correlated for boys (*r* = .88), as were the random slopes (*r* = .74). These correlations varied across ADHD subdimensions.

Correlations between random intercepts of ADHD subdimensions and the neurodevelopmental spectrum were consistently very large for inattention (*r* = .83), hyperactivity/impulsivity (*r* = .82), and cognitive disengagement (*r* = .75). Correlations between random slopes of ADHD subdimensions and the neurodevelopmental spectrum ranged from large inattention (*r* = .39) to very large for cognitive disengagement (*r* = .67).

**Girls.** The random intercepts for ADHD and the neurodevelopmental spectrum were moderately correlated (*r* = .43), so were random slopes (*r* = .74). The random intercepts and slopes of the ADHD subdimensions were each very strongly correlated with the corresponding neurodevelopmental parameters.

Correlations between random intercepts of ADHD subdimensions and the neurodevelopmental spectrum were consistently very large for inattention (*r* = .64), hyperactivity/impulsivity (*r* = .49), and cognitive disengagement (*r* = .70). Similarly, correlations between random slopes of ADHD subdimensions and the neurodevelopmental spectrum were very large (*r*s = .84 for inattention and hyperactivity/impulsivity; *r* = .64 for cognitive disengagement).

**Evidence for the Dynamic Mutualism Across Sexes**

***Externalizing Spectrum***

**Boys.** Cross-lagged associations whereby earlier ADHD prospectively predicted the externalizing spectrum (range of *B*s: -.06 to .06) and vice versa (range: -.01 to .12) were all very small to small. These findings held across ADHD subdimensions (ranges: -.05 to .11 [inattention]; -.02 to .12 [cognitive disengagement]).

**Girls.** Cross-lagged associations whereby earlier ADHD prospectively predicted the externalizing spectrum (range of *B*s: .01 to .15) and vice versa (range: -.08 to .07) were all very small to small (Table 3; Figure 5). These findings held across ADHD subdimensions (range: -.08 to .12 [inattention]; -.16 to .11 [cognitive disengagement]).

***Neurodevelopmental Spectrum***

**Boys.** Cross-lagged associations in which earlier ADHD prospectively predicted the neurodevelopmental spectrum (range of *B*s: -.18 to .10) and vice versa (range: -.14 to .07) were uniformly very small to small (Table 3; Figure 5). This pattern held across ADHD subdimensions (ranges: -.11 to .10 for inattention; -.07 to .04 for hyperactivity/impulsivity). In contrast, cognitive disengagement showed some larger cross-lagged associations with the neurodevelopmental spectrum (range: -.20 to .14), although these effects were sporadic (i.e., at three-year follow-up only).

**Girls.** Cross-lagged associations in which earlier ADHD prospectively predicted the externalizing spectrum (range of *B*s: -.22 to .11) and vice versa (range: -.15 to .00) were very small to medium in magnitude (Table 3; Figure 5). Across ADHD subdimensions, associations ranged from very small to small (ranges: -.17 to .08 for inattention; -.07 to .19 for hyperactivity/impulsivity). As in boys, cognitive disengagement showed larger cross-lagged associations with the neurodevelopmental spectrum (range: -.37 to .14), although these effects were sporadic (i.e., at three-year follow-up only).

**Evidence for Either Common Cause or Dynamic Mutualism**

***Externalizing Spectrum***

**Boys.** Within-person cross-sectional associations between ADHD and externalizing were very large (*B*s = .47 to .66). Inattention showed large to very large associations with the externalizing spectrum (*B*s = .30 to .48), whereas cognitive disengagement showed medium to large associations (*B*s = .29 to .38) with externalizing.

**Girls.** Within-person cross-sectional associations between ADHD and externalizing were very large (*B*s = .46 to .52). Inattention exhibited medium to large associations with the externalizing spectrum (*B*s = .27 to .33), and cognitive disengagement showed very small to large associations (*B*s = .02 to .30) with externalizing.

***Neurodevelopmental Spectrum***

**Boys.** Within-person cross-sectional associations between ADHD and the neurodevelopmental spectrum ranged from medium to very large and decreased over time (*B*s = .42 at baseline to .23 at three-year follow-up). This trend held for ADHD subdimensions (baseline to three-year follow-up: inattention, .52 to .04; hyperactivity/impulsivity, .38 to .23; cognitive disengagement, .39 to -.27).

**Girls.** Within-person cross-sectional associations between ADHD and the neurodevelopmental spectrum ranged from small to very large and decreased over time (*B*s = .43 at baseline to .17 at three-year follow-up). This pattern was observed across ADHD subdimensions (baseline to three-year follow-up: inattention, .25 to .11; hyperactivity/impulsivity, .32 to .20; cognitive disengagement, .39 to -.24).

1. It should be noted that models between hyperactivity/impulsivity and the externalizing spectrum did not converge. [↑](#footnote-ref-1)
